# Highlights of the 17th St Gallen International Breast Cancer Conference 2021: customising local and systemic therapies

**DOI:** 10.3332/ecancer.2021.1236

**Published:** 2021-05-18

**Authors:** Tomás Reinert, Alessandra Borba Anton de Souza, Guilherme Parisotto Sartori, Fernando Mariano Obst, Carlos Henrique Barrios

**Affiliations:** 1 Oncoclínicas, Avenida Praia de Belas 1212, CEP 90110-000, Porto Alegre, Brazil; 2 Centro de Pesquisa da Serra Gaúcha (CEPESG), Rua Vinte de Setembro 2304, CEP 95020-450, Caxias do Sul, Brazil; 3 Latin American Cooperative Oncology Group (LACOG), AV Ipiranga 6689, CEP 90619-900, Porto Alegre, Brazil; 4 Oncology Research Group-CNPq, Pontifical Catholic University of Rio Grande do Sul, Av Ipiranga 6681, CEP 90610-000, Porto Alegre, Brazil; 5 Programa de Pós-Graduação em Ciências Médicas, UFRGS, Rua Ramiro Barcellos 2400, CEP 90035-003, Porto Alegre, Brazil; 6 Hospital Sao Lucas, PUCRS, Av Ipiranga 6681, CEP 90619-900, Porto Alegre, Brazil

**Keywords:** breast cancer, 17th St Gallen Consensus Conference 2019, adjuvant, neoadjuvant, consensus

## Abstract

The 17th edition of the St Gallen International Breast Cancer Conference was held in March 2021 in an entirely virtual mode. More than 3,300 participants took part in this important bi-annual critical review of the ‘state of the art’ in the multidisciplinary care of early-stage breast cancer (BC). Seventy-four experts from all continents discussed and commented on the previously elaborated consensus questions as well as numerous interrogations on early-BC diagnosis and treatment asked by the audience. The theme of this year’s Conference was ‘Customising local and systemic therapies’. This paper summarises the results of the 2021 international panel votes as a quick news update. We discuss the most important issues on genetics, pathology, surgery, radiotherapy and systemic therapies presented and debated throughout the conference. We selected the topics based on applicability into the personalised care of BC patients and focused on questions that have a clear impact on our current clinical practice.

## Introduction

The 17th St Gallen International Breast Cancer Conference was held between 17 and 21 March 2021. Due to the COVID-19 pandemic, this edition was conducted in an entirely virtual mode. More than 3,300 participants from over 94 countries joined the live scientific sessions and the consensus meeting. Around 70 experts (detailed in Table 1) from all continents discussed and commented on an extended set of thoughtfully prepared questions as well as numerous questions on early-breast cancer (BC) diagnosis and treatment asked by the live audience. Some changes were introduced in the Conference’s chairmanship and the consensus panel composition, includ ing 21 new panelists since 2017. The panel was composed of 37 men and 22 women and included 34 medical oncologists, 13 surgeons, four gynaecologists, three radiation oncologists, two pathologists, one statistician and two public health specialists.

The objective of this 17th edition was to highlight the ‘state of the art’ of the most efficient multi-modal primary treatment of women with early BC and to focus on debating controversial issues. The theme of this year’s Conference was ‘Customising local and systemic therapies’. Notably, the consensus is based on experts’ opinions, and voting is directed to the majority of cases in a specific situation. Overall, the consensus promoted an excellent discussion of the current treatment management of BC and pointed out directions in those not so well-established questions in routine clinical care.

All the on-demand lectures had been pre-recorded, and the Consensus session closed the event. Besides, time-sensitive live discussion sessions served to debate issues and questions arising following the on-demand sessions. During the European School of Oncology (ESO) Umberto Veronesi Memorial Lecture, the award recipient, Fatima Cardoso (Champalimaud Clinical Center, Lisbon/Portugal), eloquently discussed current and future de-escalation approaches for selected patients with early-stage BC (eBC). David Cameron (Edinburgh University, Scotland) presented the Aron Goldhirsch Memorial Lecture on adjuvant therapies’ evolution across time and the future of cooperative academic clinical research groups. The 2021 St Gallen International Breast Cancer Award Lecture was ministered by Philip Poortmans (Institut Curie, France) on radiotherapy’s (RT’s) contribution to personalised care of early BC patients. The St Gallen Consensus Panel Session was split up into two sessions over the weekend – considering participants joining from all over the world with different time zones. Eric Winer (Dana Farber Cancer Institute, Boston/USA) skillfully moderated the sessions and remarkably adapted to the new virtual format.

Although clinical trial data provide the best evidence for general recommendations on clinical decision-making, data from prospective clinical research does not cover all controversies that arise when treating individual patients. Thus, expert opinion has to be used when we lack data. This is the unique feature of the St Gallen International Consensus. In making recommendations, it should be considered that the guidance applies to the vast majority of cases while acknowledging that patients’ preferences and individual situations will cause a minority of cases to fall outside of general guidance and that not all tests and treatments are available across the world. Additionally, it was assumed that the recommendations are for tumours of at least 1 cm in size (unless stated otherwise) and are applicable for patients in reasonably good general health, with a life expectancy higher than 10 years and with no medical, psychological or social circumstances that would preclude standard treatment for BC.

This paper summarises the results of the 2021 international panel votes as a quick news update. We do not intend to substitute the upcoming complete and official manuscript that will be published in the next few months.

### Genetics

The Consensus panel started with a discussion on genetic testing. Most of the panel continued not supporting genetic testing for all women with BC or all women with BC under 65 years. A risk of more than 10% for a hereditary mutation in algorithms based on family history, age at diagnosis and tumour subtype was chosen for 77% of the panel as the basis to offer genetic testing panels for hereditary cancer to women with eBC. If a gene panel testing is chosen, the majority (67%) voted that the preferred panel should routinely include: *BRCA1, BRCA2, ATM, BARD1, BRIP1, CDH1, CHEK2, NBN, PALB2, PTEN, STK11, RAD51C and RAD51D, and TP53.* A minority (7%) voted that only BRCA1 and BRCA2 should be tested, and 17.2% of the panelists opted for the evaluation of BRCA1/2 and PALB2. The panel was evenly divided concerning whether we have enough data to justify prophylactic mastectomy in patients with a *PALB2* mutation. It is important to consider that even though there are no specific studies on the issue of risk-reducing surgery in PALB2 mutation carriers, a recent publication showed a lifetime risk of developing BC of 53% in this setting [1].

Considering the recent press release about the positive results of the OlympiA trial evaluating olaparib in the adjuvant setting (to be presented at ASCO 2021), 56% of the panel voted to offer genetic testing panels for hereditary cancer to all patients who would be potential candidates to be included in this trial if results are clinical compelling. This consideration will be associated with important challenges in the near future as an increase in the demand for genetic testing and counselling will probably occur [2]. Finally, the last questions regarding genetic predisposition testing were about advising a healthy woman on optimal strategies for BC prevention versus surveillance based on gene penetrance and age. Prophylactic mastectomy was recommended only for cases with mutations in high penetrance genes (odds ratio (OR) > 3 *BRCA1, BRCA2, PALB2*), with higher support (84%) for women younger than 40 and less enthusiasm (46%) for older women (>60 years). For moderate-penetrance genes (OR 2–3 *BRAD1, CHEK2, CDH1, TP53*), independently of age, and in cases of <40-year-old patients with mutations in lower penetrance genes, most of the panel voted for breast surveillance, including MRI. Routine breast surveillance was indicated for lower penetrance genes (OR 1–2 *ATM, BRIP1, NF1, RAD51C, RAD51D, FANCC, STK11*) in women > 60 years of age by 64.15% of the panelists.

In summary, even though acknowledging the controversies regarding genomic predisposition testing, the panel’s position restates the importance of recognising a population with higher risk where testing should be mandatory. At the same time, even though the overall cost of testing has significantly declined, we still have significant barriers to overcome. Among those, physician education on predisposition management and genetic counselling availability is probably the most important. According to the panel, high- and moderate-penetrance genes should be the ones tested. Importantly, as a very clear message, the indication of bilateral mastectomies as a risk reduction procedure is a complex decision that should only be considered for specific cases after genetic counselling and multidisciplinary discussion.

### Pathology

The first question on the pathology section concerned Ki67 testing, and 62% of the panelist endorsed the recommendations from the recent publication of the International Ki67 Breast Cancer Working Group [3], suggesting that in patients with estrogen receptor (ER) + human epidermal growth factor receptor-2 (HER2) − T1-2N0-1 BC, a tumour with a Ki67 < 5% would not warrant chemotherapy, while a Ki67 > 30% would justify chemotherapy. Despite several well-known caveats, 61% of the panelists recommended that Ki67 should be part of the routine pathology report in all cases. Approximately one-third of the panelists voted for considering Ki67 only where chemotherapy may be considered, and genomic signatures assays are not available. Finally, 9% of the panelists considered that Ki67 should not be part of the routine pathology evaluation.

Regarding the use of multigene signatures to define chemotherapy indication, the panel recommended that they be considered for selected patients with ER+ HER2− BC, measuring 1–3 cm in size. These patients are generally healthy, potential candidates for chemotherapy, both pre- and postmenopausal and node-negative. In patients with 1–3 positive lymph nodes (LNs), these tests can be helpful in selected cases. In patients with four or more involved LNs, 78% of the panel members said they would never recommend genomic testing since the indication of chemotherapy is already made based on clinical features. The vast majority (92%) recommended against routine testing for program cell death-1/program cell death ligand-1 (PD1/PD-L1) in early-stage triple-negative BC (eTNBC). Regarding the routine evaluation of tumour-infiltrating lymphocytes (TILs), 61% of panelists were against it, whereas 39% recommended it. It is important to consider that even though TILs quantification has been consistently associated with prognosis, their role as a predictive biomarker remains uncertain [4].

Overall, concerning pathology, the panel signals a more formal evaluation of Ki67, particularly after the recent Working Group publication [3]. At the same time, it addressed the selection of patients that could benefit from multigene prognostic tests, particularly after the recent presentation of the Rxsponder trial data dealing with 1–3 LN-positive patients. Of note, considering that BC is the most common tumour globally and while these tests can help clinical management in selected patients, it is important to acknowledge that due to lack of access issues, most patients will continue to be managed according to clinicopathological criteria in an informed discussion with their physicians. Biomarkers for immunotherapy in eBC (PD-L1, TILs) remain in need of further validation [5].

### Imaging

In selected patients candidates for breast conservation and receiving neoadjuvant therapy for HER2-positive or TN BCs, most panelists (49%) recommend an MRI as standard baseline evaluation. In comparison, 33% would recommend it regardless of the extent of breast surgery. These responses reflect the very different practices we see across patient care scenarios across different regions in the world. The role of post-excision mammography after breast conservation surgery (BCS) was discussed, and the majority (57%) of the panelists stated that it could be avoided if excision-specimen X-rays confirm excision of all microcalcifications.

### Ductal carcinoma in situ

Initial considerations about ductal carcinoma in situ (DCIS) addressed which patients with ER-positive DCIS and adequate resection margins could avoid radiation after BCS. The panelists suggested that this approach could be considered in elderly patients and tumours with a low-grade or low-risk biological profile. There was no consensus regarding the optimal endocrine therapy (ET) to prevent recurrence, and the voting was divided among the different alternatives. Standard-dose tamoxifen or aromatase inhibitors (AIs) were considered acceptable options for 35% of the panel, while 17% recommended no ET in patients also receiving RT. These decisions should necessarily be conducted on a case-by-case basis considering the risks and benefits of ET and several features such as age, comorbidities, initial mammographic findings and biological features (especially grade) of the DCIS.

### Neoadjuvant therapy

More than a decade ago, the regulatory agencies proposed pathologic complete response (pCR) as a surrogate endpoint for drug approval in eBC. However, while the response to the neoadjuvant treatment is recognised as a prognostic biomarker, its value to approve new drugs or regimens has been the subject of several criticisms. Based on the available experience, 83% of the panelists stated that neoadjuvant pCR is encouraging, but ‘standard’ regimens should only be defined based on long-term event-free survival (EFS) and overall survival (OS) endpoints. The underlying message of the consensus states that well beyond pCR, we need studies that value EFS and OS endpoints in their design. Current and future studies should address this unmet need.

In a very clear recommendation, postmenopausal patients with low-grade and/or low-genomic signature ER-positive, HER2-negative tumours with an indication for neoadjuvant treatment, the vast majority (98%) of the panel voted in favour of neoadjuvant ET (NET) [6]. Most panelists (73%) considered that genomic assays, where available, could be performed on the core biopsy specimen to aid in this decision.

In the neoadjuvant setting of HER2-positive BC, the panel addressed the standard treatments and the post-neoadjuvant therapy for stages II and III. After the results of the TRAIN-2 study [7], much discussion has focused on which patients can be spared of anthracyclines. For 85% of the panelists, anthracyclines are not needed for stage II node-negative patients receiving taxane-based chemotherapy and anti-HER2 antibodies. It should be emphasised that this decision should be considered in the setting of access to dual anti-HER blockade with pertuzumab and trastuzumab. However, there was no real consensus for node-positive disease, where 54% of panelists voted that anthracyclines should remain part of the standard treatment with taxanes and anti-HER2 antibodies. Importantly, it should be recognised that TRAIN-2 is not the first evidence for the substitution of anthracyclines in the treatment of HER2 positive BC. Previous data from the BCIRG-006 [8] and APT [9] trials, just to mention a couple of examples, clearly point in that direction avoiding the long-term associated cardiac and haematological toxicity. Having said that, it is also essential to consider that chemotherapy without anti-HER2 therapy is curative in more than 60% of patients with HER2-positive disease, and in those circumstances, anthracyclines are an important part of the treatment. Sparing patient of anthracyclines implies in offering ideal anti-HER2 therapies as a central part of the treatment regimen.

In the neoadjuvant treatment of TNBC, there is an ongoing debate about the role of platinum salts as a standard of care in an unselected population. The addition of carboplatin to standard neoadjuvant chemotherapy (NACT) improved pCR rates without clear benefits on survival outcomes and with the cost of higher short-term haematological toxicity as seen in recent randomised clinical trials and meta-analysis [10, 11]. There was no consensus regarding the use of carboplatin with standard ‘doxorrubicin/cyclophosphamide and paclitaxel’ or other cyclophosphamide, anthracycline, taxane-type regimens, with a slight majority of panelists voting against its addition (60%). This vote reflects the current controversy on the best endpoint of these trials while defining the ideal treatment regimen for these patients. Forthcoming data on the Brightness trial’s [12] EFS will undoubtedly add important data to this discussion.

Immunotherapy emerged as a new therapeutic modality and is approved in combination with chemotherapy for first-line treatment of metastatic TNBC overexpressing PDL-1. Several ongoing trials are being conducted in the early-stage setting, and this topic has been comprehensively reviewed elsewhere [13]. Even though there are promising results showing increased pCR rates with atezolizumab and pembrolizumab, we still need to wait for mature data on survival outcomes, especially considering the risk–benefit balance with the use of immunotherapy in this setting, given the potential long-lasting immune-related adverse effects and also financial toxicity issues. The panel discouraged (90%) the use of PD1/PDL1 targeted treatment with an immune checkpoint inhibitor in the adjuvant and neoadjuvant settings and clearly defined that PD1/PDL1 testing does not help the management of eTNBC patients.

### Adjuvant systemic therapy after neoadjuvant treatment

pCR is a common efficacy endpoint in neoadjuvant therapy trials for TNBC and HER2-positive disease and has been accepted as a surrogate endpoint for drug approval. Several studies have shown that pCR is strongly associated with improved long-term survival outcomes, including progression-free survival (PFS) and OS [14]. It is clear that patients treated with neoadjuvant treatment who achieve pCR have a favourable prognosis, whereas patients who do not achieve pCR have a higher risk of relapse and should be treated differently in the adjuvant setting, as recent studies showed survival benefits with the use of trastuzumab-emtansine (TDM1) and capecitabine for patients with residual disease after neoadjuvant therapy in HER2-positive and TNBC, respectively.

Another important concept is that pCR is not a synonym of cure [15]. The majority of the experts believe that baseline stage and tumour subtype matter to define prognosis in patients achieving a pCR. Even though 34% of the panelists voted that prognosis is similar for all patients achieving pCR, we agree with the majority of the voters considering that there is solid evidence pointing out that young age, node-positive and high-grade tumours significantly increase the risk of recurrence after NACT [16]. It is known that a not-insignificant residual rate of recurrence (10%–15%) has been demonstrated in patients achieving pCR in various clinical trials.

In this regard, several questions addressed the importance of the clinical stage at presentation (before neoadjuvant therapy) to define further therapy depending on the response. In patients with HER2-positive tumours with clinically node-positive disease that achieve a pCR, the panel endorsed the use of dual HER2 blockade with pertuzumab and trastuzumab as standard adjuvant therapy. In those circumstances, for 55% of the panelists, pertuzumab must be added to trastuzumab irrespective of the initial stage, while 22% would only add pertuzumab for tumours stage III at baseline. For those patients that were initially node-negative and achieved pCR, trastuzumab alone was endorsed by 69% of the voters. In patients with residual invasive disease after standard neoadjuvant treatment, the panel endorsed the use of adjuvant trastuzumab emtansine, even for patients with low volume (<5 mm) residual invasive disease based on the pivotal KATHERINE trial [17].

In patients with TNBC and residual disease, it was considered that capecitabine is a standard of care based on the CREATE-X trial results that demonstrated both PFS and OS benefits in this context [18].

Recommendations about systemic adjuvant therapy in patients that underwent NET were discussed, even though there is clearly a paucity of evidence-based data in this field. Information from initial staging and endocrine-sensitivity during the neoadjuvant treatment should be taken into consideration in this setting. For patients treated with NET who had a good clinical response with node-negative residual cancer and favourable prognosis according to the PEPI score criteria [6], the panel unanimously discouraged the indication of adjuvant chemotherapy. However, the use of chemotherapy in the post-NET setting was strongly favoured in cases with four or more positive LNs but not necessarily in cases with a low residual disease burden in the axilla. The majority of the panelists also encouraged the consideration of adjuvant chemotherapy in patients treated with NET who had adverse prognostic factors at baseline (such as high-grade and/or intermediate-range genomic signature) as well as in cases that did not achieve considerable tumour response (for example, in ypT3).

As the indication of the neoadjuvant approach becomes part of most guidelines and is increasingly utilised, the issues of stage at presentation, adequately staging the axilla, reporting the residual cancer burden, the need for reassessing receptor expression in the residual disease, the prognostic value of the response and the development of better biomarkers or combination of biomarkers to escalate or de-escalate therapy will become much more important in patient management and trial research design.

### Management of the axilla after neoadjuvant therapy

The results of ongoing prospective trials addressing the optimal management of the axilla after NACT are eagerly awaited [19]. Considerable controversies are still seen in clinical practice given the absence of prospective data on survival outcomes after RT or axillary lymph node dissection (ALND) in this setting. Considering the volume of the residual disease in the axilla, the majority of the panel (73%) voted that ALND should be indicated following NACT when there is residual cancer in the sentinel lymph node biopsy (SNB) if macrometastasis (>2 mm) and if at least one positive out of three SNB evaluated (72%). However, in the setting of micrometastasis or isolated tumour cells (ITCs), 60% and 89%, respectively, voted that axillary dissection (ALND) can be avoided.

There was no consensus on whether a patient with cytologically/histologically proven cN1 at presentation with a good clinical response and that will receive RT could avoid an ALND if the clipped node is present in excised sentinel nodes. The majority of the panel (as well as the voting from the audience) reiterated the endorsement of ALND as standard for biopsy-confirmed cN1 patients with no or minimal clinical and radiological response**.** Considering a different scenario, for patients who present with clinically negative axilla, axillary RT was considered as a valid alternative to replace ALND in selected patients with residual disease in SNB for most of the panel: 62% said yes, if 2 of 3 sentinel lymph nodes (SLNs) were positive (at least 1 with macrometastasis), 52% said yes if 1 of 3 SNB with macrometastasis and 72% said yes if 1 of 3 with micrometastasis. One commentary after this session raised the issue that even if the axilla is clinically negative at presentation, we still do not have randomised trials to support the replacement of ALND by RT in pN+. Ninety percent (90%) supported that targeted ALND is an option in cN1 patients with clipped/marked initially involved node who convert to cN0 before surgery, and 85% voted that targeted axillary dissection is not an option exclusively in patients with favourable tumour subtype. At the end of this session, some of the panelists reiterated the importance of the multidisciplinary approach in these cases and that the radiation-oncologist should always be included in axillary management decisions after neoadjuvant therapy.

### Breast surgery

In general, the questions regarding surgery were related to de-escalation in particular situations. For women with ipsilateral breast recurrence more than 5 years after initial surgery and RT, the panel was in favour (63%) of BCS with re-irradiation compared to mastectomy. Low-risk small luminal A and long disease-free interval from initial diagnosis were considered factors that could determine BCS for ipsilateral breast recurrence for most of the panel (81% and 64%). In patients in whom re-irradiation is not an option, there was no consensus if that determines or not conservative surgical management in case of a local recurrence. For patients with a recurrence and negative nodes on imaging after previous BCS and SNB, four alternatives were presented for managing the axilla: no axillary surgery, SNB without frozen section, SNB with frozen section and ALND. Although divided, most of the panel members voted for SNB without frozen section (34%). Concerning the influence age has in these situations, according to 41% of the panel, axillary surgery could be avoided in patients > 80 years old in cases cN0. For a third of the panel members, that would be appropriate for patients > 70. A particular question addressed the morbidity of axillary surgery, and 84% of the panelists believed that preservation of the intercostal-brachial nerves should be included in the standard surgical technique.

The omission of surgery in cases of clinical and radiological complete response after neoadjuvant therapy is a subject of ongoing research and should not be used as part of routine clinical practice [20].

In summary, many questions in the surgical management of BC remain in need of randomised data. Particularly those addressing the surgical approach to the axilla after neoadjuvant therapy. Ongoing trials are addressing the specific value of radiation versus ALND. While the voting results clearly show controversial answers to the selected questions, it is essential to consider that the panel comprised a heterogeneous mix of specialties with only 13 surgeons and three radiation oncologists. As multidisciplinary management has been proposed as the best approach for BC patients, specific knowledge and interpretation of the available evidence depend on each physician’s particular area of interest. Ultimately, the voting results clearly indicate that further research efforts are required in this particular area of controversy.

### Radiation therapy

The preferred radiation treatment dose and schedule after BCS with negative margins were defined as standard hypofractionation whole-breast irradiation. Moderate hypofractionation (15–16 fractions irradiation) was considered standard of care in breast/chest wall and regional nodal irradiation (RNI) for most cases except in rare circumstances of re-irradiation and patients with very high-risk by stage and subtype for some panelists. Regarding the use of ultra-short course (five fractions) whole-breast irradiation (WBI) as used in the FAST-FORWARD trial [21], 8% considered this regimen as the preferential after BCS with negative margins in stage I–II disease, 19% considered that both options are interchangeable but 72% considered that standard hypofractionated 15–16fr WBI remains the preferential schedule.

Partial breast irradiation was not considered an appropriate option for most panelists regardless of lobular histology, hereditary gene mutation, lymphovascular invasion and age. Additionally, commercially available genomic signatures should not be used to guide decisions about RNI, postmastectomy irradiation (postmastectomy radiation therapy (PMRT)) and omission of RT in selected cases.

Regarding the indications of RNI, the panelists maintained the concept that initial staging should always be used to guide this decision. For example, in patients with cN+ receiving neoadjuvant therapy, radiation therapy should be considered regardless of the achievement of pCR in all cases for 65% of the panelists and only in initial stage III disease for 30%. On the other hand, patients presenting with cN0 disease who achieve pCR after NACT have no indication of RNI independent of being HER2-positive or TN. Patients with TNBC or ER+/HER2− T2N0 do not require PMRT and/or RNI as well.

The panelists recommended that boost should be routinely given after lumpectomy and radiation therapy for invasive BC. However, the panel was divided regarding the ideal candidates since 20% recommended the use of boost for all patients, 1% for women less than age 40, 28.8% for women less than age 50 and 31.1% recommended boost only if grade 3 or TNBC/HER2-positive subtypes regardless of age. The use of boost was not recommended routinely for DCIS, especially for low-grade lesions. Some panelists (64.7%) recommended boost for DCIS in high-risk cases (e.g., necrosis, close margins < 2 mm, larger lesions).

There was no consensus about the ideal type and timing for breast reconstruction for women likely to have PMRT. However, for patients with indication of PMRT after immediate reconstruction, the majority (%) voted that moderately hypofractionated schedules can be used without restriction.

Several questions addressed the context in which radiation therapy may be reasonably omitted after BCS for ER+HER2− tumours in older patients (>70 years) with a >10 years life expectancy. Even though some panelists voted that this option should not be considered for any subset of patients, most (88%) voted that this could be considered for patients with tumours < 2.5 cm and with low-int grade/low-genomic score.

Overall, RT discussions did evolve into the efficacy of hypofractionation schemes aligned with the recent literature showing the benefits of these shorter regimens. Clearly, there is a need to identify clinical and pathological characteristics that will allow further selection of patients for RT. Furthermore, treatment of the axilla remains a critical focus of research, both in patients treated initially with surgery and particularly in those managed with neoadjuvant therapies. The choice between ALND and RT still requires more information forthcoming in currently ongoing trials.

### Adjuvant therapy – ER-positive HER2-negative

There was no consensus about the optimal threshold to define ER expression positivity for recommending adjuvant ET in hormone receptor (HR) + BC tested by immunohistochemistry. The panel was evenly split since 50% voted for greater than or equal to 1% while the other half voted for greater than or equal to 10%. This definition remains unclear and has several practical implications not only for clinical practice but also regarding the inclusion criteria in clinical trials both in HR-positive as well as in TNBC trials. While administering ET in a patient with low HR expression is a reasonable approach, much more important, in our opinion, is that these patients should be strongly considered for chemotherapy.

Regarding the size threshold for initiating adjuvant ET in node-negative HR+ tumours, 59% of the panel members considered ET for all tumour sizes (including micro-invasive lesions), while 21% considered using ET in tumours larger than 1 mm.

For node-positive HR-positive BC, ET’s optimal duration was voted as 10 years by 53%, 7–8 years by 34% and 5 years by 11%. The panel recommended the use of ovarian function suppression (OFS) in premenopausal patients with stage II tumours, especially in patients younger than 40 years [22]. For high-risk premenopausal patients who receive 5 years of OFS plus tamoxifen, 90% of the panelists recommended the use of extended ET. Of these, 44% would recommend additional 5 years of tamoxifen monotherapy, and 40% would recommend the use of AIs (+OFS if still premenopausal). Premenopausal patients with HR-positive HER2-negative BC of sufficient risk to warrant adjuvant chemotherapy should also receive OFS if they remain premenopausal after chemotherapy. There was no consensus about the need for routine measurement of oestradiol levels during OFS.

Regarding the use of cyclin dependent kinase 4/6 (CDK4/6) inhibitors in early-stage BC, the panel was split on the indication of adjuvant abemaciclib for high-risk ER-positive, HER2-negative LN-positive BC based on the recently published monarchE trial [23].

The panel endorsed the use of the results from the MINDACT [24], RxPONDER [25] and TAILORx [26] trials to withhold chemotherapy indication in postmenopausal patients with low-risk signatures and/or recurrence equal or inferior to 25. However, concern was raised by half of the panelists regarding the use of genomic signatures in patients with high-risk tumours such as pT3N1 or patients with more than three positive nodes, since in these settings, the use of adjuvant chemotherapy should be recommended regardless of the genomic tests’ results.

Potential differences regarding the clinical utility of genomic tests between premenopausal and postmenopausal patients have been a matter of great interest recently [27]. Differently than for postmenopausal patients, the RxPONDER trial showed that premenopausal patients with node-positive BC had a reduction in the risk of recurrence with the use of adjuvant chemotherapy. A similar situation was seen in the subgroup of the TAILORx trial comprising premenopausal patients with node-negative disease and an intermediate level recurrence score. The question remains as to whether this is a direct benefit of chemotherapy, or an indirect effect of ovarian suppression. When questioned about the recommended treatment for premenopausal women with node-negative BC and recurrence score of 16–25 or other low-range genomic signature, 22% voted for tamoxifen, 24% for chemotherapy and ET, while the majority (53%) choose the option of OFS combined with tamoxifen or an AI.

The panel was divided when asked about the recommended treatment or premenopausal patients with 1–3 positive LNs and recurrence score up to 25 (or other low-range genomic signature). Chemotherapy and oral ET were chosen by 30%, OFS and oral ET by 17%, 26% stated that either treatment approach is reasonable but favours chemotherapy. In contrast, the same proportion of 26% of the panelists voted that either treatment approach is reasonable but favours ET.

Addressing the optimal chemotherapy regimen in stage II ER-positive BC, most panelists voted that any ‘standard’ anthracycline/cyclophosphamide/taxane-based protocol is acceptable, whereas 44% voted to use TC for four or six cycles. In stage III HR+ BC, more than 90% indicated the use of anthracyclines and taxanes. Interestingly, the traditional cyclophosphamide-methotrexate-fluoracil (CMF) combination had no votes in this discussion.

Overall, the panel stressed the controversy on the definition of HR-positive disease, an important subject that remains without a definitive answer. Most of the panel suggested the need for adjuvant therapy for minimal invasive disease. In a very clear vote, the panel gave importance to OFS in the management of premenopausal patients, particularly those with high-risk characteristics. However, the ideal duration of ET in these patients remains an unanswered question. Finally, the use of prognostic genomic signatures was validated by the panel with our caveat of their limited availability in most of the world, where clinicopathological factors, an informed discussion and patient preference will continue to be the basis for treatment recommendations.

### Adjuvant therapy – HER2-positive and TNBC

OS is the gold standard endpoint for randomised clinical trials in the adjuvant setting. However, several caveats and difficulties in demonstrating OS benefits in clinical research, such as lack of statistical power and post-progression therapies that may dilute the benefits associated with the adjuvant therapies, led to the use of surrogate endpoints for drug approval. This topic was brought into discussion in the context of the Olympia trial (NCT 02032823) that will be presented at this year´s ASCO Meeting. When questioned about what is the magnitude of survival benefit necessary to recommend adjuvant olaparib for BC patients with germline BRCA1/2 mutation, most panelists (48%) chose an absolute invasive disease free-survival (iDFS) benefit at 3 years of 5%, whereas 8% voted that would only recommend adjuvant Olaparib if OS advantage is demonstrated.

Regarding the size threshold for initiating adjuvant anti-HER2 therapy in node-negative HER2-positive tumours, 51% of the panel members considered the size of 5 mm, while 22.6% recommended the threshold of 6 mm. Curiously, approximately one-fourth of the panelist suggested the use of anti-HER2 therapy for pT1a and even micro-invasive tumours. Even though this is an arbitrary decision, we acknowledge that there is no clinical evidence for anti-HER2 therapy in pT1a tumours, and we recommend adopting pT1b as the size cut-off in parallel with international guidelines.

Consistent with the updated analysis from the APPHINITY trial [28], 94% of the panel recommended that patients with node-negative HER2-positive tumours should not receive adjuvant pertuzumab. The use of weekly paclitaxel plus trastuzumab is the standard of care for stage I HER2-positive BC based on results from the APT trial [29]. Using TDM1 in this setting, based on results from the ATTEMPT (study), should only be considered as an alternative in exceptional circumstances.

Regarding the size threshold for considering adjuvant chemotherapy in stage I TNBC, the cut-off of 5 mm was the most voted (%). There is no data to justify the use of immune checkpoint inhibitors in the adjuvant setting for TNBC outside the context of a clinical trial, as previously discussed in the neoadjuvant treatment section.

### Oligometastatic breast cancer

Even though the St Gallen Conference is devoted to eBC, the topic of oligometastatic BC was also discussed with interest. While the exact definition of oligometastatic disease is debatable, according to the advanced breast cancer-5, this situation refers to low-volume metastatic disease with a limited number and size of metastatic lesions (up to five and not necessarily in the same organ), potentially amenable to local treatment at achieving a complete remission status [30]. The panel discussed a hypothetical situation of a patient with clinical stage T2N1 BC that presented with *de novo* mBC to the sternum (single site); most panelists (84%) recommended therapy with curative intent with optimal systemic and local therapy (radiation treatment to include the sternal lesion) in managing this case. Probably important as well, whether the tumour subtype influences decision-making in this context remains unclear.

### Survivorship and quality of life

Four major domains should be addressed in BC survivors: a recurrence and a new primary cancer, long-term and late effects, modifiable health behaviours and care coordination. A few of these issues were addressed in questions presented to the panel. In women with significant genitourinary or sexual health symptoms on adjuvant ET not alleviated with moisturisers or lubricants, 88% of the panelists would commonly recommend intravaginal oestrogens to relieve symptoms. However, most experts (72%) would also explain to the patient that there is no scientific-based confidence about safety with this practice. Cold-caps could be routinely offered to patients receiving chemotherapy that induce alopecia for 69% of the voters. Most panelists (80%) agreed that meditation, particularly mindfulness-based stress reduction, be recommended to alleviate depressive symptoms for BC patients. Concerning health behaviours, alcohol consumption should be limited to an average of one drink per day, according to 57% of the voters. Aerobic exercises are endorsed by 43% as a standard approach.

## Conclusion

The tradition of the St Gallen Consensus Conference continues. Unquestionably, the need to address controversial issues and point the direction of future research is an essential attribute of the event and what generates the most attention. As mentioned, the composition and expertise of the panel do have an important but not quantifiable influence in many of the questions that address some particular subjects that sometimes require a very detailed and careful interpretation of the literature.

Despite these caveats, we believe the Conference has the ability to bring some of the issues to an authentic scenario where clinicians from many different regions of the world and practicing in very diverse scenarios do manifest the same doubts.

## Conflicts of interest

**Tomás Reinert**

- Speaker honoraria: Novartis, AstraZeneca, Pfizer, Libbs, Lilly, Roche
- Consulting or advisory role: AstraZeneca, Lilly, Novartis
- Research funding: AstraZeneca, Libbs

**Alessandra Borba Anton de Souza:** none

**Guilherme Parisotto Sartori:** none

**Fernando Mariano Obst:** none

**Carlos Henrique Barrios**

- Stock and other ownership interests: Biomarker, MedSIR, Tummi
- Speaker honoraria: Novartis, Roche/Genentech, Pfizer, GlaxoSmithKline, Sanofi, Boehringer Ingelheim, Eisai
- Consulting or advisory role: Boehringer Ingelheim, Roche/Genentech, Novartis, GlaxoSmithKline, Eisai, Pfizer, AstraZeneca, Libbs, MSD Oncology, United Medical
- Research funding: Pfizer, Novartis, Amgen, AstraZeneca, Boehringer Ingelheim, GlaxoSmithKline, Roche/Genentech, Lilly, Sanofi, Taiho Pharmaceutical, Mylan, Merrimack, Merck, AbbVie, Astellas Pharma, Biomarin, Bristol-Myers Squibb, Daiichi Sankyo, Abraxis BioScience, AB Science, Asana Biosciences, Medivation, Exelixis, ImClone Systems, LEO Pharma, Millennium, Janssen, Atlantis Clinica, INC Research, Halozyme, Covance, Celgene, inVentiv Health
- Travel, accommodations, expenses: Roche/Genentech, Novartis, Pfizer, BMS Brazil, AstraZeneca, MSD Oncology

## Funding statement

There was no funding for this manuscript.

## Figures and Tables

**Table 1.** Participants of the 17th St Gallen Breast Cancer Conference 2021.

**Conference founder and honorary chair**
Hans-Joerg Senn, c/o Tumor and Breast Center ZeTuP, St Gallen/Switzerland
**Conference Co-chairs**
Michael Gnant, Medical University of Vienna/Austria; Beat Thürlimann, Breast Center St Gallen, Kantons-spital St Gallen/Switzerland
Scientific programme-chairpersons
Harold Burstein, Boston/MA, USA
Giuseppe Curigliano, Milano/Italy
Michael Gnant, Vienna/Austria
Meredith Regan, Boston/MA, USA
Hans-Jörg Senn, St Gallen/Switzerland
Beat Thürlimann, St Gallen/Switzerland
Walter Weber, Basel/Switzerland
Eric Winer, Boston/MA, USA
**Panelists**
Aapro Matti (Switzerland)
Aebi Stefan (Switzerland)
André Fabrice (France)
Barrios Carlos (Brazil)
Bergh Jonas (Sweden)
Bonnefoi Herve (France)
Bretel Morales Denisse (Peru)
Brucker Sara (Germany)
Burstein Harold (United States)
Cameron David (United Kingdom)
Cardoso Fatima (Portugal)
Carey Lisa (United States)
Cescon Dave (Canada)
Chua Boon (Australia)
Ciruelos Eva (Spain)
Coates Alan (Australia)
Colleoni Marco (Italy)
Cortes Javier (Spain)
Curigliano Giuseppe (Italy)
Delaloge Suzette (France)
Denkert Carsten (Germany)
Dubsky Peter (Switzerland)
Ejlertsen Bent (Denmark)
Fitzal Florian (Austria)
Francis Prudence (Australia)
Galimberti Viviana (Italy)
Gamal Heba (Egypt)
Garber Judy (United States)
Giordano Sharon (United States)
Gnant Michael (Austria)
Gradishar William (United States)
Gulluoglu Bahadir (Turkey)
Harbeck Nadia (Germany)
Heil Jörg (Germany)
Huang Chiun-Sheng (Taiwan)
Huober Jens (Switzerland)
Ilbawi Andre (WHO Cancer Control Programme)
Jiang Zefei (China)
Johnston Steven (United Kingdom)
King Tari (United States)
Lin Nancy (United States)
Loi Sherene (Australia)
Loibl Sibylle (Germany)
Morrow Monica (United States)
Krop Ian (United States)
Lee Eun Sook (Korea)
Pagani Olivia (Switzerland)
Partridge Ann (United States)
Piccart Martine (Belgium)
Poortmans Philip (Belgium)
Prat Aleix (Spain)
Pusic Andrea (United States)
Regan Meredith (United States)
Rubio Isabella (Spain)
Rugo Hope (United States)
Rutgers Emiel (Netherlands)
Sedlmayer Felix (Austria)
Semiglazov Vladimir (Russian Federation)
Shao Zhiming (China)
Spanic Tanja (Europa Donna)
Tesarova Petra (Czech Republic)
Thürlimann Beat (Switzerland)
Tjulandin Sergei (Russian Federation)
Toi Masakazu (Japan)
Trudeau Maureen (Canada)
Turner Nicholas (United Kingdom)
Tutt Andrew (United Kingdom)
Vaz Luis Ines (France)
Viale Giuseppe (Italy)
Watanabe Toru (Japan)
Weber Walter (Switzerland)
Winer Eric (United States)
Xu Binghe (China)
